# Glyphosate and Triton X-100 single-competitive sorption–desorption analysis through kinetic and equilibrium frameworks utilizing maximum likelihood method

**DOI:** 10.1007/s11356-025-37082-z

**Published:** 2025-11-10

**Authors:** Hamid Moghimi, Mohaddeseh Mousavi Nezhad, Marijke Huysmans

**Affiliations:** 1 Porous Materials and Processes Modelling Research Group, School of Engineering, the University of Warwick, Coventry, CV4 7AL UK; 2 Department of Civil and Environmental Engineering, School of Engineering, the University of Liverpool, Liverpool, L69 3GH UK; 3 Department of Water and Climate, Vrije Universiteit Brussel (VUB), Pleinlaan 2, 1050 Brussels, Belgium

**Keywords:** Glyphosate, Groundwater contamination, Kinetic-equilibrium analysis, Maximum likelihood method, Single-competitive sorption–desorption, Triton X-100

## Abstract

This study investigates the sorption and desorption dynamics of glyphosate and Triton X-100 under both single and competitive conditions within equilibrium and kinetic frameworks. Understanding competitive sorption–desorption, determining the leaching potential, is critical in agricultural contexts where pesticides and surfactants can migrate through soil and contaminate groundwater. This study develops an advanced inverse model based on a maximum likelihood algorithm to characterize sorption–desorption, integrating single and competitive isotherms to estimate key parameters from batch experiments. The study further explores the impacts of compound competition, concentration ratios, and soil properties on sorption–desorption processes, while providing a quantitative comparison between equilibrium and kinetic frameworks. Results indicate that Triton X-100 exhibits minimal sorption with maximum sorption capacity of 0.2 mg/g_soil_, which sorption characteristics remain nearly identical regardless of soil composition or the presence of glyphosate. For glyphosate, with a maximum sorption capacity up to 27 mg/gsoil, the presence of minerals such as albite, muscovite, kaolinite, and illite can increase sorption by up to 45%, resulting in higher sorption to desorption ratio under kinetic conditions and achieving equilibrium in a shorter timeframe. Moreover, the MLM results indicate that competitive conditions reduce glyphosate sorption by up to 10%, with a further 10% decrease observed as the Triton X-100 concentration increases from 0.5 to 2%. Kinetic analysis shows that glyphosate sorption–desorption, in both single and competitive conditions, includes an initial rapid sorption phase where approximately 70% of total sorption occurs with minimal desorption, followed by a slower phase as the system nears equilibrium. The presence of Triton X-100, especially at higher concentrations, extends the rapid sorption phase and delays equilibrium, altering glyphosate’s sorption pattern. Moreover, peak sorption–desorption rates occur within the first 100 min in single-sorption scenarios, while the presence of Triton X-100 slows the sorption rate, spreading it up to 300 min.

## Introduction

Soil and groundwater contamination by pesticides and surfactants is a critical global concern (Pasquini et al. 2025). Glyphosate, one of the most extensively used pesticides, is valued for its broad-spectrum activity and non-selective action. However, due to its toxicity and leaching potential, it poses a significant risk to groundwater (Moghimi et al. 2025; Osten et al. 2025), with harmful effects on human health, particularly the nervous system (Bloem et al. 2024). Triton X-100, a common surfactant in pesticide formulations, improves performance by aiding compound transport to target areas, such as plant root zones (Graziano et al. 2023). Given the simultaneous application of these compounds, understanding their behavior in soil is essential to enhance efficacy while reducing contamination risks to soil and groundwater (Moghimi et al. 2024; Kaur et al. 2024; Karaduman and Kelleci Çelik 2024).

Competitive sorption–desorption is a key mechanism controlling the leaching behavior of pesticides and surfactants, such as glyphosate and Triton X-100, in agricultural soils. This process plays a crucial role in determining their environmental fate and potential to contaminate groundwater resources (Amini Tapouk et al. 2020; Moghimi et al. 2022; St. John Warne et al. 2023). Understanding competitive sorption–desorption necessitates a realistic framework that incorporates both equilibrium and kinetic analyses in single and binary systems. This approach is essential to accurately determine the maximum sorption capacity and sorption–desorption rates under varying conditions (Niknejad et al. 2020), and the influence of key parameters, including chemical properties (Yuan et al. 2023), soil organic and inorganic content (Jabin et al. 2021), and pesticide-to-surfactant concentration ratios (Costa et al. 2023). Quantifying single and competitive sorption–desorption mechanisms under diverse conditions requires advanced models (Yousefi et al. 2021, 2025) capable of accurately analyzing laboratory data from batch and column experiments as well as field data (Li et al. 2023). These models must operate effectively within equilibrium and kinetic frameworks, capturing distinctions between them, as kinetic view is more representative of real-world (Sanchez-Huerta et al. 2023).

Recent studies have made efforts to comprehend the sorption–desorption of glyphosate (Besghaier et al. 2021; Gairhe et al. 2021) and Triton X-100 (Wiśniewska et al. 2021) within soil and groundwater. Recent works show that factors such as the mineral composition of agricultural soils (Dotor-Robayo et al. 2022), variations in concentration across soil depths (Fenn et al. 2023), and interactions with other compounds (Ghavamifar et al. 2023) significantly influence glyphosate’s sorption–desorption dynamics. Triton X-100, known to enhance chemical transport (Jiménez-González et al. 2019) by altering sorption properties (Lu et al. 2018), has also been a focus. Studies have addressed glyphosate’s multicomponent sorption involving compounds like phosphate (Dotor Robayo et al. 2024), cadmium’s (Pattanaik et al. 2025), and calcite (Ghavamifar et al. 2023), utilizing inadequate competitive isotherm models in equilibrium (De Gerónimo And Aparicio 2022) and kinetic frameworks (Jiang et al. 2020), and insufficient assessment of kinetic variations from equilibrium conditions (Li And Jaisi 2019; Dotor-Robayo et al. 2022). For the effective utilization of isotherms in competitive sorption–desorption analysis, recent studies have developed multi-component sorption and desorption models tailored to different chemicals (Georgii 2021) under both kinetic (Jiang et al. 2020) and equilibrium conditions (Wang et al. 2018). Janetti et al. (2012) investigated competitive sorption models within an equilibrium framework, emphasizing that the effectiveness of different models depends on the specific soil types and chemicals involved. Phanikumar et al. (2022) developed two-site and three-site models to characterize contaminant interactions in aqueous and solid phases, emphasizing the significance of sorption capacity in determination of sorption behavior for various compounds. Chen et al. (2022) utilized a dual-porosity model to examine interactions between sorbed and aqueous phases and contaminant retardation, revealing kinetic differences based on isotherm parameters. Also, Georgii (2021) employed a multi-compound sorption model based on Langmuir isotherms to describe sorption under mixed-component conditions, demonstrating distinct results compared to simpler unary models. Mohammadnejad et al. (2020) developed a two-stage kinetic sorption model, emphasizing the importance of compound interactions in sorption processes.

Although single sorption–desorption of pesticides and surfactants, such as glyphosate and Triton X-100, has been studied (Azimzadeh et al. 2024), and recent research has developed multi-component sorption–desorption models for various chemicals, the competitive sorption–desorption of pesticides and surfactants, a common agricultural practice remains unexplored. Furthermore, even in multicomponent studies that include these compounds alongside other chemicals, appropriate competitive analysis that account for chemical interactions are not employed (De Gerónimo And Aparicio 2022), which the competitive effects on sorption–desorption mechanisms remain insufficiently understood. Moreover, the effects of concentration ratios between pesticides and surfactants, such as glyphosate and Triton X-100, on their interactions, maximum sorption capacity, and kinetic rates during competitive sorption–desorption in agricultural soils remain unclear (Appah et al. 2020). The development of an inverse model capable of analyzing single and competitive sorption–desorption of pesticides and surfactants within both equilibrium and kinetic frameworks is a crucial gap in this field. Such a model would enable a quantitative comparison to identify the most representative isotherms while highlighting the added value and novel insights provided by the kinetic framework for competitive sorption–desorption across various soils (Zhao et al. 2024).

This study develops an advanced inverse model based on the maximum likelihood method (MLM), integrating equilibrium and time-dependent batch sorption–desorption experimental data for glyphosate and Triton X-100, two widely used compounds in agricultural practices. The main aim of this study is to utilize advanced optimization algorithms to precisely quantify the competitive sorption–desorption behavior of glyphosate and Triton X-100 in agricultural soils under varying conditions, compare equilibrium and kinetic approaches, and emphasize the significance and added value of a kinetic framework and the time-dependent nature of sorption mechanisms. Moreover, as a novel perspective within the field, the effects of soil composition and the concentration ratio between glyphosate and Triton X-100 on single and competitive sorption–desorption are quantified. Quantifying the time-dependent variations in sorption–desorption behavior of compounds within a competitive framework, along with the development of an advanced optimization model, offers a novel and valuable perspective for characterizing sorption–desorption processes and supporting large-scale models in assessing groundwater contamination risks.

## Laboratory research

### Soil characterization

Four European agricultural soils were collected from a depth of 0–1 m and stored at − 4 °C in black bags to minimize moisture loss and microbial activity. Additionally, a silica sandy soil, characterized by low organic and inorganic content and no agricultural history, was used as a basic soil. According to the OECD standard for batch sorption experiments (Sittig et al. 2025), soil samples were air-dried at room temperature (25 °C) and then sieved (2 mm) to control the soil particles size, and the pH of the soil is measured by a pH meter (HI 991001 N, Hanna Instruments). Also, the particle density of soil samples (g/cm^3^) is measured by the GASJAR method (Smith 2021) as presented in Table 1.

**Table 1:** Physical properties of soils, used in the batch experiments

Soil	Soil A	Soil B	Soil C	Soil D	Sany soil
Particle density (g/cm^3^)	2.542	2.483	2.576	2.491	2.658
pH (-)	6.52	6.64	7.18	6.56	6.92
Organic content (%)	3.234	3.456	3.341	3.286	-

Agricultural soil components, encompassing both organic and inorganic contents, are analyzed to assess the key factors influencing the competitive sorption–desorption mechanisms. The organic content of agricultural soils is determined using the loss on ignition (LOI) method (Satoh et al. 2023) as measurement results are presented in Table 1. Also, complementary analytical techniques based on X-ray fluorescence (XRF) and X-ray diffraction (XRD) methods are applied to determine the elemental composition (Table 2) and mineralogical/crystallographic data (Table 3) of the soils qualitatively and quantitatively.

**Table 2:** XRF results for the chemical composition of agricultural soils

Component	Chemical Formulation	Fraction (%)			
		Soil A	Soil B	Soil C	Soil D
Sodium	Na	0.262	0.203	0.173	0.142
Magnesium	Mg	0.422	0.697	0.585	0.645
Aluminum	Al	6.489	6.257	6.440	6.127
Silicon	Si	45.84	41.122	34.498	41.753
Phosphorous	P	0.304	0.435	0.161	0.326
Sulfur	S	0.137	0.314	0.149	0.192
Potassium	K	8.384	9.849	8.037	10.464
Calcium	Ca	1.879	6.350	23.547	6.138
Titanium	Ti	3.359	4.459	2.607	4.735
Manganese	Mn	0.637	0.986	0.778	3.169
Iron	Fe	29.74	25.706	28.801	24.015
Zink	Zn	0.165	0.208	0.148	0.250

**Table 3:** XRD results for the mineralogy of agricultural soils

Mineral	Chemical formulation	Soil A	Soil B	Soil C	Soil D
Quartz	Sio_2_	▢	▢	▢	▢
Albite	Na Al Si_3_ O_8_	▢	-	▢	-
Microcline	K Al Si_3_ O_8_	▢	▢	-	-
Muscovite	K Al_2_(Al Si_3_ O_10_) (F, OH)_2_	▢	▢	▢	▢
Illite	(K, H_3_O) (Al Mg Fe)_2_(Si Al)_4_ O_10_[(OH)_2_ (H_2_O)]	▢	▢	-	▢
Gibbsite	Al (OH)_3_	▢	-	-	-
Kaolinite	Al_2_ Si_2_ O_5_ (OH)_4_	▢	-	▢	-
Orthoclase	K Al Si_3_ O_8_	▢	▢	-	▢
Caldecahydrite	Ca Al_2_ O_4_ (H_2_O)_10_	▢	-	▢	-
Koninckite	(Fe, Al) PO_4_ (H_2_O)_3_	▢	▢	-	-
Muscovite	K Al_2_ (Si_3_Al) O_10_(OH, F)_2_	▢	▢	-	-
Birnessite	K_0.5_ Mn_2_ O_4_ (H_2_O)_1.5_	▢	-	▢	▢
Calcite	Ca CO_3_	▢	▢	▢	▢

### Chemical characterization

Potassium bromide (KBr) (Merck, 10,031–22-8), an inorganic chemical, is used as a conservative controller for batch experiment process. Calcium chloride (Merck, 10,043–52-4) is an aqueous solvent used for preventing sedimentation in the batch experiment. All stock solutions are prepared in 0.01 Molar cacl_2_ solved in double deionized water (DDW) (prepared by Alto Type I Polisher, Triple Red, 18.2 M). Glyphosate (Merck, 1071–83-6), a non-residual pesticide, is used as target chemical in this study. According to the agricultural applications and detected contamination in the environment (Gairhe et al. 2021), experiments were operated with an glyphosate initial concentration of 25 to 400 (mg/L) for equilibrium experiments and a concentration of 100 (mg/L) for kinetic experiments. Triton X-100 (Merck, 9036–>19-5), a non-ionic surfactant with common applications in agricultural practice, is used in concentrations range of 0.1 to 2% (surfactant weight (g)/solution volume (L)) (Lin et al. 2016) for equilibrium and kinetic experiments. Concentration measurements are based on the ion chromatography–mass spectrometry (IC-MS) detection method (Valle et al. 2019). The IC–MS (Thermoscientific) is used, which was equipped with IonPack 4 μm Infinity Lab Poroshell 120 EC-C18 (4.6 × 150 mm, AGILENT TECHNOLOGIES) with basic solvent potassium hydroxide. Detailed information about the developed chromatography approach and calibration can be found in Appendix 1.

### Experimental framework

The standard batch sorption methodology based on OECD standard (Sittig et al. 2025) was used to quantify the sorption–desorption rates in single and competitive cases in equilibrium and kinetic conditions. According to the standard, a 1:5 soil/solution ratio is applied for batch experiments using 10 g of air-dried agricultural soil mixed with a 50-mL solution containing glyphosate and Triton X-100 prepared in 0.01 M calcium chloride in a DDW solution. All experiments were carried out at room temperature (i.e., a temperature ranging between 20 and 25 °C). KBr is used as a conservative controller to rule out any potential physiochemical reactions or contamination stemming from the plastic tubes, filtration system, and concentration measurement device in use. For equilibrium experiments after mixing the solution and soil in Teflon tubes, each sample shaken for 24 h at 250 rpm to ensure equilibrium condition. But for kinetic experiments, samples shake for 600 and the sampling time is each 60 min. After controlling the pH, samples were centrifuged (Sigma Compact Centrifuge) at 9000 rpm for 30 min to separate the soil particles and solution. Finally, 2 mL of each sample was filtered (GD/X 25 Syringe Filter, sterile, 0.2 µm), and chemical concentrations were measured by chromatography approach IC–MS. To evaluate the robustness of the experimental results and parameter estimation, statistical analyses were performed. All batch experiments were conducted in triplicate to quantify experimental error and ensure the validity and repeatability of the procedure, as detailed in Appendix 2, where mean values with standard deviations are reported. In addition, the uncertainty associated with the experimental data is illustrated in the “Results and discussions” section through error bars, which were calculated as the standard error of the mean and are shown in the figures.

## Model development

### Governing equations

Equilibrium sorption and kinetic sorption–desorption models are employed to quantitatively assess these mechanisms for glyphosate and Triton X-100 under both single and competitive conditions (Coutinho et al. 2021). Sorption–desorption in unary, binary, and multi-component systems are often interpreted by using linear (Urano et al. 1984), Langmuir (Matthijs And De Henau 1985), or Freundlich (Abe et al. 1985) isotherms. A kinetic model represents difference between the sorption and desorption rates *Q* (mg/g_soil_.min) and is presented as.

1 $$Q=\partial S/\partial t=\mathrm{Sorption}\;\mathrm{rate}\left(C,\;S\right)-\mathrm{Desorption}\;\mathrm{rate}\;\left(S\right)$$

which is described by linear, Freundlich, and Langmuir models as.

2 $$\mathrm{Kinetic}\;\mathrm{Linear}\;Q=k_{sor\_d}C-k_{des\_d}S$$

3 $$\mathrm{Kinetic}\;\mathrm{Freundlich}\;Q={\mathrm k}_{\mathrm{sor}\_\mathrm F}\mathrm C^{\mathrm m}-{\mathrm k}_{\mathrm{des}\_\mathrm F}\mathrm S$$

4 $$\mathrm{Kinetic}\;\mathrm{Langmuir}\;Q=K_{sor\_L}C\left(S_{max}-S\right)-k_{des\_L}S$$

In these models, *S* is the amount of the chemical component retained by the soil (mg/g_soil_) and *C* is the concentration of the component (mg/L) in the aqueous phase. In the linear model, which describes a linear pattern between sorption–desorption and concentration, the parameters *k*_*sor_d*_ (L/g_soil_. min) and *k*_*des_d*_ (1/min) are sorption and desorption constants, respectively. Freundlich model considers nonlinear behavior using nonlinearity term *m* and sorption–desorption constants *k*_*sor_F*_ (L^m^. mg^1−m^/min.g_soil_) and *k*_*des_F*_ (1/min). Langmuir model considers the maximum sorption capacity of the soil *S*_max_ (mg/g_soil_) to describe the sorption behavior with sorption–desorption constants *k*_*sor_L*_ (L/mg. min) and *k*_*des_L*_ (1/min). In the equilibrium condition, that the sorption and desorption rates are equal (i.e., *Q* = 0), so a new parameter as equilibrium sorption constant is defined K=*k*_*sor*_/*k*_*des*_, and kinetic models are simplified to equilibrium format (Janetti et al. 2012) as presented in the following:

5 $$\mathrm{Equilibrium}\;\mathrm{Linear}\;S\mathit=KC$$

6 $$\mathrm{Equilibrium}\;\mathrm{Freundlich}\;S\mathit=K_{\mathit F}C^{\mathit m}$$

7 $$\mathrm{Equilibrium}\;\mathrm{Langmuir}\;S\mathit=\mathit{\left({K_LCS_{max}}\right)}\mathit/\mathit{\left({1+K_LC}\right)}$$

In equilibrium models, *K* (L/g_soil_), *K*_*F*_ (L^m^/mg^1−m^. G_soil_), and *K*_*L*_ (L/mg) are sorption parameters for linear, Freundlich, and Langmuir isotherms, respectively. The kinetic and equilibrium sorption–desorption models for competitive conditions used in this study are developed as a multi-reaction model based on single models, which considers possible physiochemical reactions between two chemicals in a binary system, regarding their mutual effects on the sorption–desorption behavior in a competitive condition. The kinetic competitive sorption–desorption model considers three reversible simultaneous reactions between chemical species and soil, for the species to sit on the sorption sites as (Gimsing et al. 2004),

8a $$1)S_i+C_j\leftrightarrow S_j+C_i$$

8b $$2)C_j\leftrightarrow S_j$$

8c $$3)C_i\leftrightarrow S_i$$

The first reversible reaction represents the effect of one compound’s concentration on the other compound’s sorption, which captures the competition between pesticide and surfactant for free sorption sites on sorbent. The second and third reactions represent the sorption and desorption of pesticide and surfactant in a kinetic condition (Nitta et al. 1984). The developed Langmuir model for kinetic sorption–desorption considers sorbent fraction that can be occupied by both compounds, which describes the competition for free sorption sites.

9 $$Q_i=\frac{d(S_i)}{dt}=k_{sor\_i}S_{max}C_i{(1-\sum\limits_{j=1}^NS_j/S_{max})}^{\in_i}-k_{des\_i}S_i$$

10 $$Q_j=\frac{d(S_j)}{dt}=k_{sor\_j}S_{max}C_j{(1-\sum\limits_{j=1}^NS_j/S_{max})}^{\in_j}-k_{des\_j}S_j$$

In this model, *S*_*i*and*j*_ represent the sorption of chemicals at time *t*. Also, *C*_*i*and*j*_ are concentrations (mg/L) at each detection time. Following Langmuir’s single model, *k*_sor_ and *k*_des_ are sorption and desorption coefficients for components, respectively. Parameter *ϵ* represents the fraction of the sorption site occupied by sorbate molecules in the sorbed phase. For equilibrium conditions, extended Langmuir model (Tsai and Li 2008) and Sheindorf–Rebhun–Sheintuch (SRS) model (Schwarz 1978) are used to describe the sorption behavior of glyphosate, Triton X-100 in the binary experiments.

11 $$Extended\;Langmuir\;S_i=\frac{S_{max}K_{L,i}C_i}{1+\sum_{j=1}^NK_{L,J}C_j}$$

12 $$\mathrm{SRS}\;{\mathrm S}_{\mathrm i}={\mathrm K}_{\mathrm F,\mathrm i}{\mathrm C}_{\mathrm i}{(\sum_{\mathrm j=1}^{\mathrm N}{\mathrm\alpha}_{\mathrm{ij}}{\mathrm C}_{\mathrm j})\;}^{{\mathrm m}_{\mathrm i}-1}$$

where *S*_*i*_ is the amount of sorbed chemical *i* (mg/g_soil_), and *C*_*i*_ and *C*_*j*_ are the equilibrium concentrations (mg/L) of chemical species *i* and *j*, respectively. In the Langmuir model, *S*_max_ represents the saturation of all sorption sites (mg/g_soil_). In the SRS model, *α*_*ij*_ represents the competition between chemical species to land on sorption sites.

### Maximum likelihood method

**Fig. 1:**
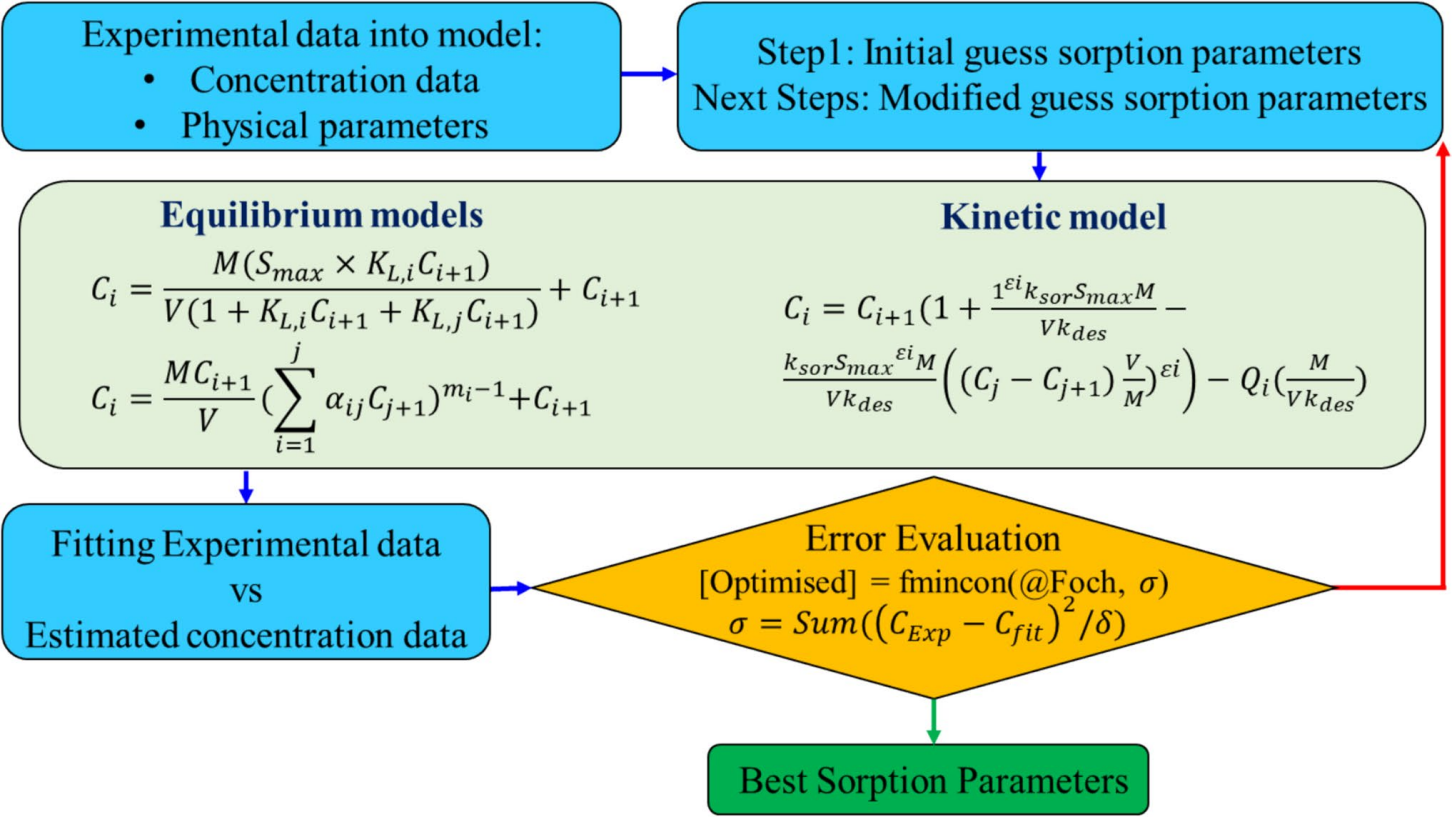
Performance overview of the developed MLM: optimization algorithm estimating parameters based on experimental data, utilizing “fmincon” function on MATLAB

In the present study, an inverse algorithm based on the maximum likelihood method is developed to estimate sorption–desorption parameters for single and competitive models in equilibrium and kinetic conditions. MLM is a statistical approach used to estimate the parameters of a probability distribution that best explains observed data. The developed model finds the best values of the sorption–desorption parameters that maximize the likelihood of the data, assuming a particular distribution (Moghimi et al. 2023a, b). In order to use MLM for parameter estimation, sorption models need to be calibrated according to concentration values. Batch kinetic and equilibrium experiments provide the concentration of pesticide or surfactant in each sampling time step. Using a second definition for the sorption parameter, it is possible to change sorption–desorption equations and rewrite them based on concentration parameters, which makes it appropriate to be used in the inverse modeling algorithm and estimate sorption and desorption parameters. Figure 1 provides an overview of the developed MLM performance, illustrating how the optimal parameters are estimated from experimental data through the applied optimization algorithm. The inverse model is developed by relying on the statistical functions (fmincon) of MATLAB, estimating values of sorption–desorption parameters for single and competitive models as well as determining their associated uncertainty.

The accuracy of the developed inverse model is verified by comparing its predicted sorption isotherms for single and competitive sorption of heavy metals on two types of soil reported by Janetti et al. (2012), which estimated sorption isotherm parameters in equilibrium conditions. As presented in Appendix 3, the estimated parameters from the two studies are highly consistent, with a reliable error rate of less than 5%. For model fitting, residual analysis was carried out to evaluate the coefficient of determination (*R*^2^ (-)) and the root-mean-square error (RMSE, expressed in both mg/L and %), in order to compare the accuracy of model predictions for equilibrium and kinetic models under both single-solute and competitive sorption conditions. All statistical analyses were performed using the statistical toolboxes available in MATLAB (Sun et al. 2022).

The main strengths of this study include well-designed batch experiments, robust data interpretation, and the development of an advanced inverse method based on an optimization algorithm, enabling comprehensive single and competitive sorption analysis within both equilibrium and kinetic frameworks. However, the study has certain limitations, including scale constraints, potential variability in soil properties, and the inherent restrictions of batch methods in simulating dynamic environmental conditions.

A batch experiment is chosen to study the sorption–desorption of glyphosate and Triton X-100, as it provides precise control over solute concentrations, solution chemistry, and soil conditions, which is essential for isolating competitive effects and obtaining reproducible equilibrium and kinetic data. The batch approach in the present study is the first stage of our research, which is subsequently extended through column experiments to capture dynamic conditions, and later to field-scale modelling within an agricultural setting. Compared with column (Moghimi et al. 2025) or field studies (Moghimi et al. 2024), batch tests minimize hydraulic variability and focus on soil-solution interactions. Additionally, the inverse modelling approach, utilizing a maximum likelihood algorithm, is applied to integrate single and competitive isotherms, enabling the robust estimation of sorption–desorption parameters. This combination of batch experiments and inverse modelling provides a reliable and quantitative framework to analyze competitive sorption processes.

This research involves potential risks that should be considered. First, the use of glyphosate and Triton X-100 poses chemical handling risks due to their potential environmental and health impacts if not managed properly; therefore, strict safety protocols were followed throughout all laboratory procedures. Second, while the batch experiments provide valuable insights, the laboratory-scale results may not fully reflect the complexity of real field conditions, limiting direct applicability without further field validation. Finally, the numerical model developed in this study relies on several assumptions, such as homogeneous porous media and simplified sorption parameters, which may introduce uncertainties when extrapolated to heterogeneous natural systems.

## Results and discussions

### Equilibrium analysis

**Fig. 2:**
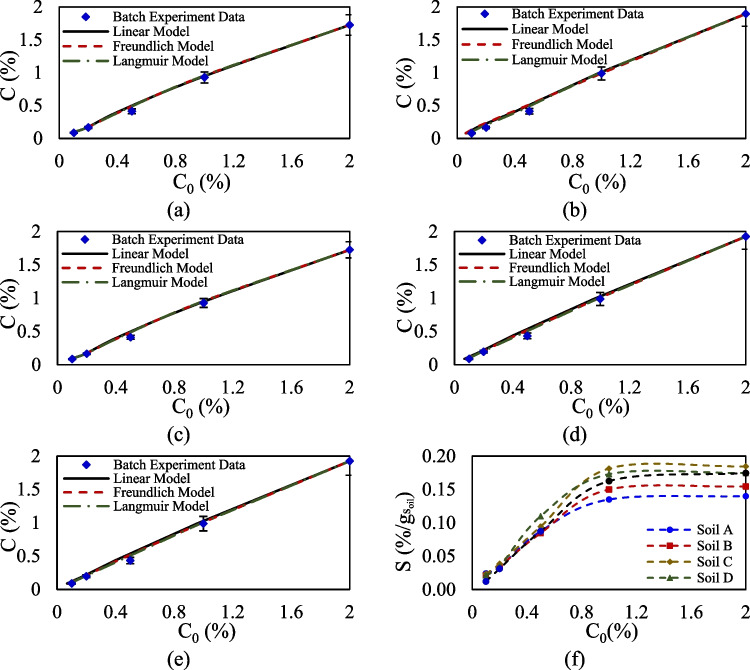
Experimental equilibrium concentration data and calibrated models for single sorption of Triton X-100 on **a** soil A, **b** soil B, **c** soil C, **d** soil D, and **e** sandy soil. **f** Comparison of single equilibrium sorption of Triton X-100 in one gram of sorbent

**Fig. 3:**
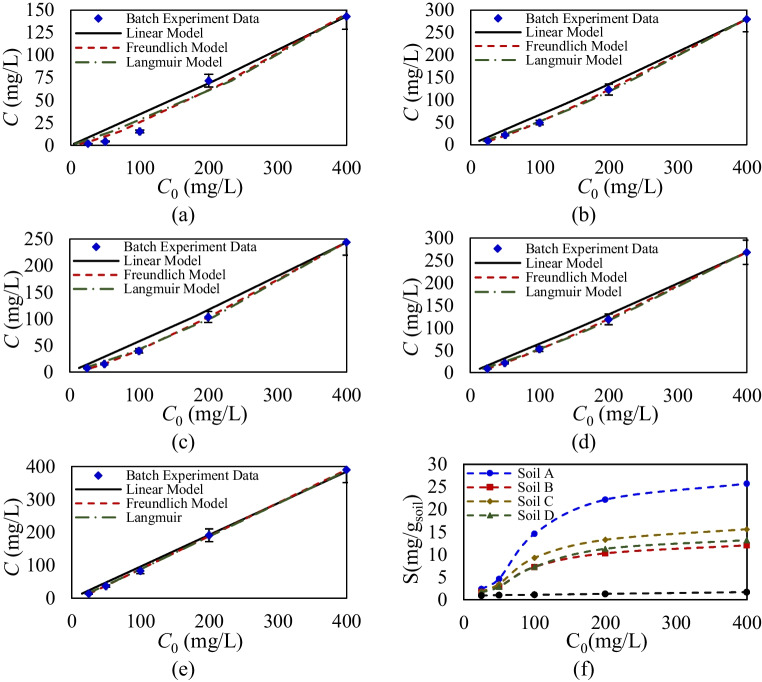
Experimental equilibrium concentration data and calibrated models for single sorption of glyphosate on **a** soil A, **b** soil B, **c** soil C, **d** soil D, and **e** sandy soil. **f** Comparison of single equilibrium sorption of glyphosate in one gram of sorbent

The analysis begins by examining the equilibrium single sorption of glyphosate and Triton X-100 across four agricultural soils and a sandy soil. The aim is to define their maximum sorption capacities, explore how these capacities vary in relation to specific soil characteristics, and evaluate the performance of the developed inverse model based on MLM for different isotherms. Figures 2 and 3 illustrate the initial and equilibrium concentrations of glyphosate and Triton X-100 across varying concentrations in batch equilibrium experiments for different soils, comparing observed sorption values against the fitted linear, Langmuir, and Freundlich models. Sorption parameters and model accuracies, determined via the developed MLM, are presented in Table 4. Error bars in each figure indicate the precision of the experimental data, with additional supporting data available in Appendix 2.

**Table 4:** Equilibrium single-sorption isotherm parameters for glyphosate and Triton X-100 estimated by MLM

Model	Soil A	Soil B	Soil C	Soil D	Sandy soil
	Glyphosate				
Linear model					
*K*_*d*_ (L/g_soil_)	3.132	1.221	2.216	1.636	0.232
*R*^2^ (-)	0.811	0.802	0.791	0.811	0.852
RMSE (mg/L)	5.343	5.156	5.457	5.325	3.237
Freundlich model					
*K*_*F*_ (L^m^/mg^1−m^. g_soil_)	2.142	1.134	1.838	1.231	0.216
*m* (-)	0.647	0.716	0.662	0.749	0.912
*R*^2^ (-)	0.861	0.863	0.885	0.870	0.921
RMSE (mg/L)	5.084	4.954	4.854	4.671	3.143
Langmuir model					
*S*_max_ (mg/g_soil_)	26.547	12.135	16.245	14.248	1.845
*K*_*L*_ (L/mg)	38.252	25.375	31.375	23.356	5.325
*R*^2^ (-)	0.936	0.936	0.948	0.943	0.954
RMSE (mg/L)	3.144	3.564	3.657	3.537	2.146
	Triton X-100				
Linear model					
*K*_*d*_ (L/g_soil_)	0.214	0.192	0.181	0.191	0.111
*R*^2^ (-)	0.853	0.865	0.857	0.837	0.894
RMSE (%)	0.148	0.159	0.184	0.152	0.158
Freundlich model					
*K*_*F*_ (L^m^/mg^1−m^. g_soil_)	0.194	0.172	0.165	0.183	0.132
*m* (-)	0.914	0.923	0.887	0.931	0.919
*R*^2^ (-)	0.925	0.921	0.924	0.931	0.912
RMSE (%)	0.162	0.147	0.165	0.174	0.185
Langmuir model					
*S*_max_ (mg/g_soil_)	0.165	0.187	0.197	0.174	0.138
*K*_*L*_ (L/mg)	4.115	2.116	2.614	2.313	0.511
*R*^2^ (-)	0.956	0.967	0.961	0.976	0.961
RMSE (%)	0.169	0.183	0.189	0.174	0.168

Comparing the sorption behavior for Triton X-100 across different soils in Fig. 2a–e indicates that this surfactant exhibits minimal sorption capacity in all tested agricultural soils and sandy soil. This observation suggests that Triton X-100 is weakly retained by soil particles, regardless of variations in soil mineralogy and composition, which typically influence sorption behaviors in more reactive compounds. Figure 2 f reinforces this finding, showing consistently low sorption capacities across soil types, confirming that Triton X-100’s interaction with soil is relatively uniform and limited. Furthermore, the data reveal that after reaching a concentration threshold of approximately 1.2%, the sorption levels of Triton X-100 plateau, meaning that further increases in the compound’s concentration do not result in a corresponding rise in sorption. This is attributed to saturation of the limited binding sites available for Triton X-100 within the soil or to a lack of strong chemical affinities between the compound and soil.

The results presented in Fig. 3a–e indicate distinct sorption characteristics for glyphosate across different agricultural soils. Glyphosate exhibits significantly higher sorption values in these soils, following a nonlinear sorption behavior that aligns well with Langmuir isotherm model (Ayenew And Getu 2025). This nonlinear behavior suggests that glyphosate’s sorption is influenced by soil properties, including mineral composition, which vary across soil types in this study. Also, Fig. 3f presents the comparison of glyphosate sorption values in one gram of silica sandy soil and agricultural soils across different concentrations, which reveal significant differences. Also, this figure clearly presents the nonlinearity of sorption for glyphosate at different concentrations. Soil A demonstrates the highest sorption capacity, suggesting strong interactions between glyphosate molecules and soil constituents that enhance retention. In contrast, sandy soil, which lacks substantial organic content and key minerals, shows markedly lower glyphosate sorption capacity, highlighting its limited ability to retain this compound. Soils B and D also exhibit relatively lower sorption capacities compared to soils A and C, but they still surpass the values seen in sandy soil.

The variations in glyphosate’s sorption capacity and nonlinearity are attributed to differences in soil composition, including both organic and inorganic components. Based on the results of LOI and XRF/XRD analyses, the agricultural soils display similar organic content but differ significantly in mineralogical composition. Glyphosate, an organic compound with dual polarity, demonstrates a notable affinity for binding to minerals present in agricultural soils, and its sorption capacity is strongly influenced by the mineral composition (An et al. 2022). Silica sandy soil contains only quartz, known for its minimal sorption capacity. In contrast, soils A and C, which contain higher amounts of albite, illite, and kaolinite compared to soils B and D, exhibit improved glyphosate sorption capacity and nonlinearity. These minerals, known for their surface area and cation exchange properties, provide numerous binding sites for glyphosate, enhancing its retention in these soils (Guo et al. 2021). Recent studies have highlighted that minerals such as illite (Jabin et al. 2021) and kaolinite (Heryanto et al. 2025) enhance the sorption capacity of agricultural soils for various chemicals, including pesticides. Moreover, the presence of specific metal oxides and cations, such as iron, potassium, and aluminum, further amplifies glyphosate’s sorption, as established in the literature (Junqueira et al. 2020). Soil A, which exhibits the highest glyphosate sorption among the studied soils, can be directly linked to its elevated amounts of aluminum (6.249%), potassium (8.384%), and iron (29.74%), which enhance the soil’s ability to retain glyphosate, leading to prolonged travel times and higher retardation factors (Gairhe et al. 2021).

The developed MLM quantifies the equilibrium sorption behavior of glyphosate and Triton X-100 by determining sorption capacity, assessing nonlinearity, and evaluating the performance of various isotherms. As shown in Table 4, the linear model exhibits poor performance, highlighting the necessity of nonlinear isotherms for accurate representation. The evaluated nonlinearity parameter in the Freundlich model indicates that glyphosate exhibits strong nonlinear sorption behavior, particularly in soils A and C. In contrast, Triton X-100 demonstrates relatively low nonlinearity across all agricultural soils, suggesting more uniform sorption characteristics. Based on the Langmuir model, glyphosate demonstrates varying sorption capacities across different soil types. The highest sorption capacity is observed in soil A, with a maximum of 26.547 mg/g_soil_, while the lowest is found in soil B, with a value of 12.135 mg/g_soil_. In comparison, Triton X-100 exhibits a relatively consistent sorption affinity across all soil types, although it shows a considerably lower maximum sorption capacity of *S*_max_ < 0.197 mg/g_soil_. Also, model accuracy parameters show that the Langmuir model provides the highest accuracy in predicting sorption parameters.

The single-sorption equilibrium results confirm the strong sorption affinity of glyphosate compared to Triton X-100, which is consistent with its high polarity and strong interactions with soil minerals. This supports our initial hypothesis that glyphosate would dominate sorption in the absence of competing solutes. The relatively low sorption of Triton X-100 highlights its weaker binding to soil particles, which has direct implications for its higher mobility and potential leaching (Azimzadeh et al. 2024).

**Fig. 4:**
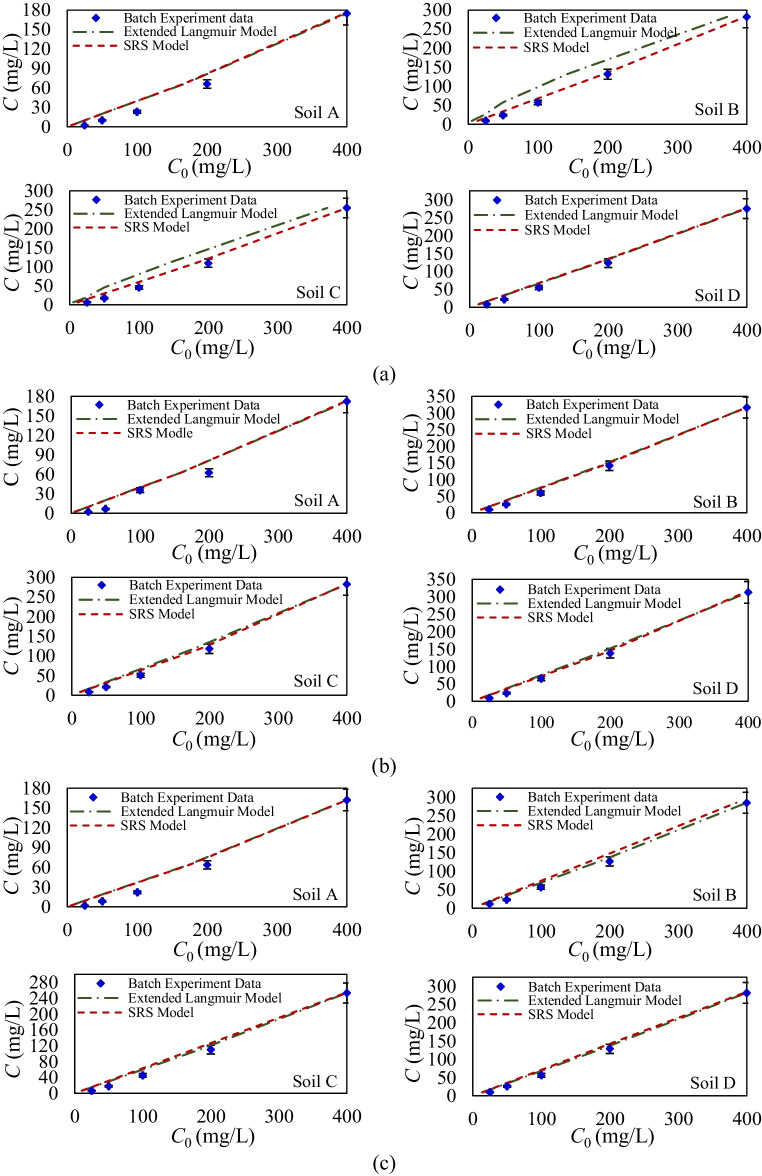
Experimental competitive equilibrium concentration data of glyphosate and calibrated models with Triton X-100 concentration of **a** 0.5%, **b** 1%, and **c** 2%

Based on the single-sorption results, the competitive sorption of glyphosate and Triton X-100 on agricultural soils was analyzed to investigate the impacts of their mutual interactions on the sorption mechanism within a binary system. Figure 4 illustrates the initial and equilibrium concentrations of glyphosate at various concentration ratios, alongside equilibrium nonlinear isotherms Extended Langmuir and SRS fitted for competitive sorption scenarios at Triton X-100 concentrations of 0.5%, 1%, and 2%. Results for Triton X-100 sorption in the presence of glyphosate are not shown, as glyphosate does not affect Triton X-100’s sorption capacity or behavior, and batch equilibrium sorption results are almost identical as single-sorption analysis. However, Triton X-100 substantially alters the sorption capacity and behavior of glyphosate. The competitive sorption analysis is further supported by the estimated sorption parameters and their accuracy, assessed via MLM, as detailed in Table 5. While the developed isotherms for competitive sorption analysis do not directly reveal the type and effectiveness of reactions involved, the sorption intensity and estimated parameters provide insight into the effective reaction characteristics of the soils and chemicals.

**Table 5:** Estimated sorption parameters for equilibrium competitive sorption of glyphosate (i) and Triton X-100 (j) for three different applied concentration of Triton X-100

	Triton X-100 0.5%			
	Soil A	Soil B	Soil C	Soil D
Extended Langmuir model				
*K*_*i*_ (L/mg)	31.677	19.442	25.554	19.483
*K*_*j*_ (L/mg)	4.016	2.015	2.016	2.015
*S*_max_ (mg/g_soil_)	22.541	11.057	14.809	12.731
*R*^2^ (-)	0.869	0.885	0.891	0.894
RMSE (mg/L)	4.654	4.545	4.445	4.428
SRS model				
*K*_*i*_ (L^m^/mg^1−m^. g_soil_)	1.637	1.129	1.531	1.011
*K*_*j*_ (L^m^/mg^1−m^. g_soil_)	0.184	0.193	0.181	0.169
*m*_*i*_ (-)	0.650	0.813	0.683	0.819
*m*_*j*_ (-)	0.963	0.953	0.966	0.937
*α*_*ij*_ (-)	0.994	0.978	0.988	0.996
R^2^ (-)	0.842	0.824	0.813	0.835
RMSE (mg/L)	5.134	5.243	5.648	5.348
	Triton X-100 1%			
Extended Langmuir model				
*K*_*i*_ (L/mg)	27.476	18.267	22.545	16.343
*K*_*j*_ (L/mg)	4.014	2.013	2.015	2.016
*S*_max_ (mg/g_soil_)	21.198	10.215	12.197	11.648
*R*^2^ (-)	0.863	0.876	0.863	0.871
RMSE (mg/L)	4.366	4.154	4.445	4.238
SRS model				
*K*_*i*_ (L^m^/mg^1−m^. g_soil_)	1.431	1.021	1.338	0.965
*K*_*j*_ (L^m^/mg^1−m^. g_soil_)	0.185	0.189	0.184	0.165
*m*_*i*_ (-)	0.667	0.842	0.715	0.884
*m*_*j*_ (-)	0.981	0.926	0.969	0.984
*α*_*ij*_ (-)	0.986	0.954	0.956	0.915
R^*2*^ (-)	0.841	0.852	0.838	0.839
RMSE (mg/L)	5.167	5.156	5.323	5.287
	Triton X-100 2%			
Extended Langmuir model				
*K*_*i*_ (L/mg)	24.248	16.342	19.218	14.402
*K*_*j*_ (L/mg)	4.015	2.015	2.016	2.014
*S*_max_ (mg/g_soil_)	20.015	8.549	11.219	8.826
*R*^2^ (-)	0.876	0.874	0.879	0.869
RMSE (mg/L)	4.321	4.245	4.254	4.334
SRS model				
*K*_*i*_ (L^m^/mg^1−m^. g_soil_)	1.228	0.919	1.029	0.913
*K*_*j*_ (L^m^/mg^1−m^. g_soil_)	0.184	0.179	0.183	0.164
*m*_*i*_ (-)	0.741	0.902	0.797	0.895
*m*_*j*_ (-)	0.993	0.935	0.968	0.982
*α*_*ij*_ (-)	0.972	0.977	0.983	0.992
*R*^2^ (-)	0.842	0.838	0.851	0.836
RMSE (mg/L)	5.254	5.523	5.165	5.355

Competitive sorption results indicate that while glyphosate has minimal impact on the sorption capacity and nonlinearity of Triton X-100, the presence of Triton X-100 significantly reduces the sorption capacity of glyphosate and alters its sorption behavior across all agricultural soils. This reduction suggests that at high concentrations of Triton X-100 in the aqueous phase, even minimal sorption of this surfactant affects the availability of active soil binding sites for glyphosate. Triton X-100 alters glyphosate’s sorption behavior by modifying the physicochemical properties of soil surfaces (Appah et al. 2020) and interfering with the binding sites glyphosate would typically occupy. As a result, the soil’s affinity for glyphosate decreases, as Triton X-100 competes with or inhibits glyphosate from accessing these sites, ultimately reducing the maximum sorption capacity of glyphosate in soils where Triton X-100 is present. Comparing the competitive sorption effects of Triton X-100 on glyphosate to prior competitive studies on heavy metals (Padilla et al. 2023), pharmaceuticals (Zhang et al. 2022), etc., highlights a substantial difference in the extent of reduced sorption capacity observed for the target chemical. For other substances, given the comparable concentration ranges, the coexistence of two or more compounds tends to moderately decrease the sorption of each compound. But for the studied pesticide and surfactant, the presence of Triton X-100 leads to a more pronounced reduction in glyphosate sorption capacity in all agricultural soils. This intensified effect resonates with the practical use of surfactants, aiming to diminish pesticide sorption.

The MLM effectively quantifies competitive sorption parameters and identifies variations compared to single-component scenarios, providing insights into how glyphosate and Triton X-100 influence each other’s sorption capacity and nonlinearity. The results indicate that both competitive models accurately represent the sorption behavior of these compounds. However, the extended Langmuir model exhibits slightly higher accuracy (*R*^2^ > 0.869, RMSE < 4.654) compared to the SRS model (*R*^2^ > 0.813, RMSE < 5.648). This superior performance is attributed to the extended Langmuir model’s ability to better account for the maximum sorption capacity of the soils, making it a more reliable choice for modeling competitive sorption in agricultural soils. The sorption parameters derived from both models reveal that the nonlinearity terms and sorption capacities for Triton X-100 are nearly identical to those determined under single-sorption conditions.

This suggests that Triton X-100 exhibits non-influencing behavior in competitive scenarios, with its sorption unaffected by the presence of glyphosate. In contrast, models’ quantifications indicate that the presence of Triton X-100 significantly impacts glyphosate’s sorption parameters. According to the SRS model, the nonlinearity term decreases across all agricultural soils in the presence of varying concentrations of Triton X-100. Model results indicate that Triton X-100 can reduce nonlinearity terms by up to 20% compared to single-component sorption conditions. Furthermore, the extended Langmuir model reveals that even at its lowest concentration, Triton X-100 causes a significant 15% reduction in the maximum sorption capacity of glyphosate under competitive conditions relative to single-sorption scenarios. MLM results confirm Triton X-100 alter the physicochemical properties of soil, potentially modifying surface charge, disrupting bonding, or occupying active sorption sites that glyphosate would otherwise utilize. As a result, glyphosate experiences reduced affinity for soil particles, leading to lower sorption capacity and a shift toward more linear behavior.

**Fig. 5:**
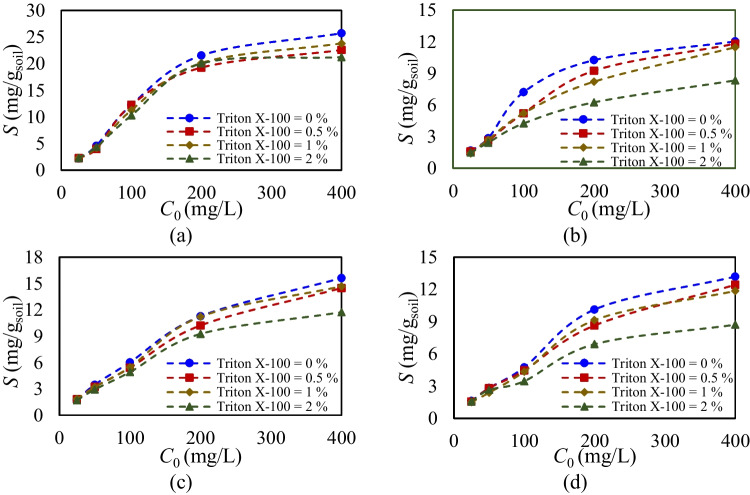
Sorption amount of glyphosate for different concentrations of Triton X-100 (0%, 0.5%, 1%, and 2%) per gram of agricultural soils. **a** Soil A, **b** soil B, **c** soil C, and **d** soil D

As shown in Fig. 4 and Table 5, different concentration ratios of glyphosate and Triton X-100 were analyzed to assess their impact on glyphosate sorption in a binary system, helping to clarify the glyphosate interaction in the presence of the surfactant. Here, Fig. 5 shows the amount of glyphosate (mg) sorbed per gram of agricultural soil at different Triton X-100 concentrations (0%, 0.5%, 1%, and 2%). The results indicate that increasing Triton X-100 concentrations consistently reduce glyphosate sorption capacity across all agricultural soils, following a similar trend but with varying intensities. Specifically, increasing Triton X-100 from 0.5 to 2% results in sorption declines of 17%, 10%, 25%, and 30% for soils A, B, C, and D, respectively. This reduction occurs as Triton X-100 interacts with soil components, enhancing competition and displacing glyphosate from sorption sites. While soils A and C experience significant reductions in sorption capacity but retain high nonlinearity, soils B and D exhibit both substantial decreases in capacity and reduced sorption nonlinearity. These findings, corroborated by MLM results, underscore the combined influence of concentration ratios and soil composition in shaping the extent of Triton X-100’s interference with glyphosate sorption. The equilibrium analysis clarifies the competitive interactions between compounds and the impact of concentration ratios between the pesticide, surfactant, and soil composition. However, it does not account for the temporal dynamics of these variations, highlighting the need for a time-dependent perspective.

Under competitive conditions, a clear reduction in glyphosate sorption was observed, particularly at higher concentrations of Triton X-100, indicating direct competition for sorption sites. This outcome supports the initial concept that surfactants can displace or inhibit pesticide retention, thereby enhancing the potential for glyphosate transport. The slight increase in Triton X-100 sorption in the presence of glyphosate suggests secondary interactions with organic matter or surface modification effects, emphasizing the complexity of multi-solute systems (Ghavamifar et al. 2023).

### Kinetic analysis

**Fig. 6:**
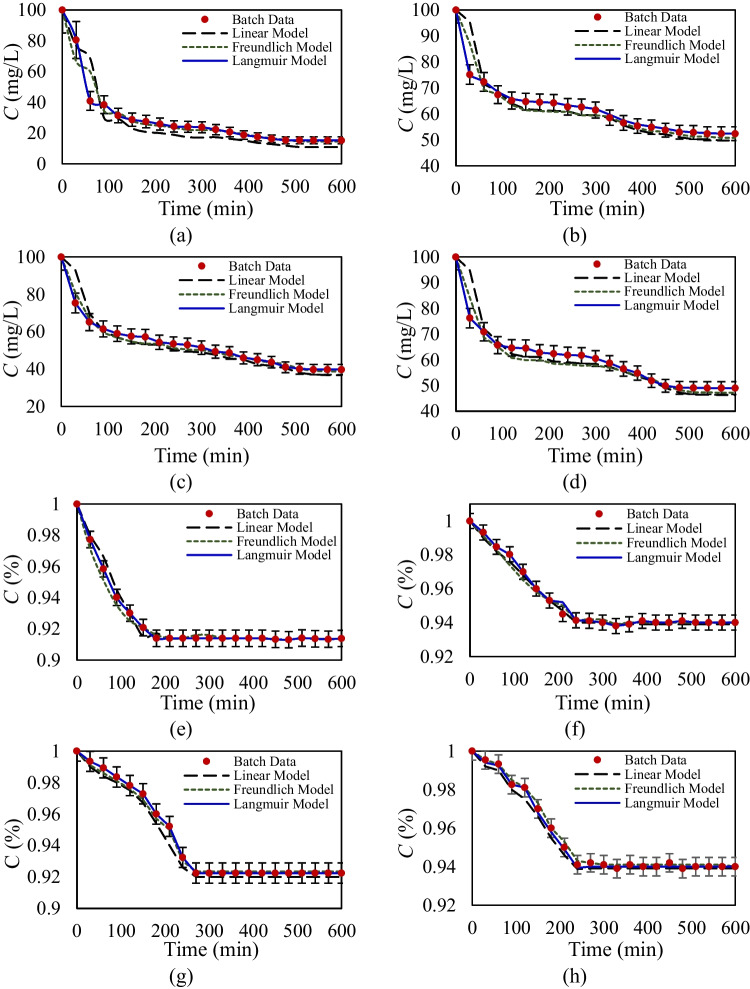
Concentration variation over time and calibrated models for kinetic single-sorption–desorption for glyphosate: **a** soil A, **b** soil B, **c** soil C, and **d** soil D, and Triton X-100: **e** soil A, **f** soil B, **g** soil C, and **h** soil D

The inclusion of kinetic sorption–desorption analysis offers a temporal perspective on the interactions between glyphosate and Triton X-100 within agricultural soils. This temporal view allows for a more nuanced understanding of how these substances behave over time, providing insights into the dynamic nature of the sorption and desorption and understanding the rate at which equilibrium is achieved. The kinetic analysis begins by evaluating the single-sorption–desorption behavior of glyphosate and Triton X-100 on agricultural soils, using initial concentrations of 100 mg/L for glyphosate and 1% for Triton X-100. Figure 6 illustrates the concentration variations of glyphosate and Triton X-100 over a 600-min period, highlighting the sorption–desorption process. The results are analyzed using calibrated isotherms, including linear, Freundlich, and Langmuir kinetic models, to describe the underlying sorption mechanisms. Furthermore, Table 6 provides the sorption–desorption parameters estimated using the developed MLM, offering detailed insights into the sorption kinetics and behavior for both compounds.

**Table 6:** Kinetic single-sorption–desorption isotherms parameters for glyphosate and Triton X-100 estimated by MLM for different agricultural soils

Glyphosate					Triton X-100			
	Soil A	Soil B	Soil C	Soil D	Soil A	Soil B	Soil C	Soil D
Linear model								
*k*_*sor_d*_ (L/g_soil_. min)	0.087	0.037	0.065	0.048	0.006	0.004	0.005	0.004
*k*_*des_d*_ (1/min)	0.043	0.014	0.032	0.023	0.004	0.003	0.004	0.003
*R*^2^ (-)	0.748	0.769	0.749	0.773	0.818	0.819	0.821	0.823
RMSE (mg/L)	3.347	3.326	3.653	3.364	0.173	0.175	0.163	0.139
Freundlich model								
*k*_*sor_F*_ (L^m^. mg^1−m^/min.g_soil_)	0.055	0.028	0.039	0.031	0.004	0.003	0.004	0.003
*k*_*des_F*_ (1/min)	0.033	0.016	0.027	0.020	0.003	0.002	0.002	0.002
*m* (-)	0.804	0.810	0.728	0.822	0.968	0.969	0.988	0.977
*R*^2^ (-)	0.827	0.831	0.829	0.835	0.865	0.874	0.861	0.874
RMSE (mg/L)	2.654	2.578	3.175	3.003	0.159	0.153	0.158	0.157
Langmuir model								
*k*_*sor_L*_ (L/mg. min)	0.849	0.419	0.647	0.397	0.117	0.149	0.138	0.122
*k*_*des_L*_ (1/min)	0.253	0.167	0.217	0.148	0.108	0.119	0.124	0.137
*S*_max_ (mg/g_soil_)	22.15	9.154	13.24	11.15	0.257	0.248	0.253	0.234
*R*^2^ (-)	0.913	0.921	0.919	0.924	0.943	0.939	0.952	0.927
RMSE (mg/L)	2.157	2.987	2.217	2.397	0.134	0.138	0.141	0.146

Single-sorption–desorption kinetic data for Triton X-100 indicates minimal sorption capacity across all agricultural soils, with equilibrium conditions being reached rapidly, approximately 250 min after the start of the kinetic experiment. In contrast, glyphosate exhibits strong sorption across agricultural soils, with sorption capacity following the order: soil A > soil C > soil D > soil B, consistent with equilibrium data, where sorption of glyphosate ranges from 50 to 80%, influenced by the mineralogical composition of the soils. The data indicates that glyphosate reaches equilibrium at a slower rate compared to Triton X-100, requiring approximately 480 min from the start of the kinetic experiment. The distinct kinetic sorption behaviors of Triton X-100 and glyphosate arise from their differing chemical properties. Triton X-100 exhibits minimal sorption due to weak interactions with soil, while glyphosate, a polar compound with a strong affinity for soil particles, undergoes complex binding mechanisms, and requires more time to reach equilibrium during kinetic experiments. Also, equilibrium observations indicated that higher mineral content enhances the sorption capacity of agricultural soils for glyphosate. Complementing these findings, kinetic results reveal that soils with higher mineral content not only exhibit stronger glyphosate sorption but also achieve equilibrium more rapidly due to increased sorption rates. The greater mineral content in soils A and C contributes to a more interconnected pore structure, increasing the available surface area for glyphosate interaction, thereby providing more active binding sites and accelerating sorption rates and equilibrium attainment.

The single kinetic MLM results for glyphosate and Triton X-100 indicate that the Freundlich model captures the highly nonlinear sorption–desorption behavior of glyphosate, whereas Triton X-100 exhibits nearly linear sorption kinetics. The Langmuir model effectively determines maximum sorption capacities, aligning with equilibrium findings. A key advantage of kinetic models is their ability to quantify both sorption and desorption rates, providing insight into how rapidly these compounds interact with soil particles. Based on statistical parameters, the Langmuir model best represents the sorption–desorption dynamics (*R*^2^ > 0.913), particularly for glyphosate (RMSE < 2.987), which exhibits high sorption capacity and nonlinearity. In contrast, the linear model performs poorly, and the Freundlich model is not ideal for accurately describing glyphosate’s kinetic behavior. In the kinetic Langmuir model, Triton X-100 exhibits nearly equal sorption and desorption rates, indicating its low overall sorption capacity due to weak interactions with soil particles. In contrast, glyphosate shows significantly higher sorption rates compared to desorption rates, particularly in soils with greater sorption capacity. This difference is primarily attributed to soil composition and mineralogy, which enhance glyphosate’s binding strength and retention within the soil matrix.

The kinetic results for single-solute experiments reveal that glyphosate sorption reached equilibrium more rapidly and with a higher overall capacity than Triton X-100. This finding aligns with the hypothesis that glyphosate has stronger site-specific binding, leading to faster sorption kinetics. By contrast, the slower uptake of Triton X-100 reflects diffusion-limited processes and weaker site interactions, reinforcing its higher persistence in the aqueous phase.

**Fig. 7:**
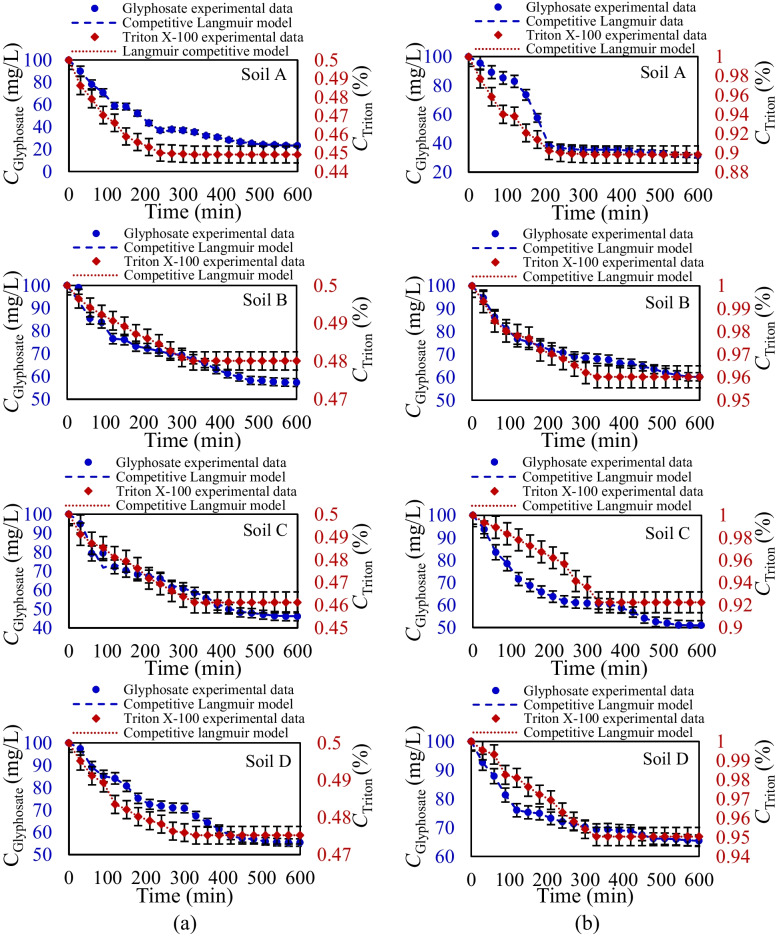
Glyphosate and Triton X-100 concentrations variation over time with calibrated model (competitive Langmuir model) for kinetic competitive sorption–desorption experiments applying Triton X-100 concentrations of 0.5% (**a**) and 1% (**b**)

Building on the findings from competitive equilibrium analysis and single kinetic analysis, the study further examines the kinetic sorption–desorption behavior of glyphosate and Triton X-100 under competitive conditions. Figure 7 presents the concentration variations of glyphosate and Triton X-100 under competitive conditions over a 600-min period. In these cases, glyphosate is maintained at an initial concentration of 100 mg/L, while Triton X-100 is introduced at initial concentrations of 0.5% and 1% to evaluate the impact of concentration ratios on glyphosate’s sorption–desorption rates. Also, Table 7 presents the competitive Langmuir isotherm parameters and sorption–desorption values estimated using MLM for both competitive scenarios for glyphosate and Triton X-100.

**Table 7:** Kinetic competitive sorption-desorption parameters for glyphosate (i) and Triton X-100 (j) estimated by MLM, Langmuir model for different Triton X-100 concentrations

Triton X-100 0.5%									
	*K*_sor, i_ (L/mg. min)	*K*_des, j_ (1/min)	*ϵ*_i_ (-)	*K*_sor, j_ (L/mg. min)	*K*_des, j_ (1/min)	*ϵ*_j_ (-)	*S*_max_ (mg/g_soil_)	R^2^ (-)	RMSE (mg/L)
Soil A	0.732	0.262	0.761	0.132	0.107	0.239	19.147	0.887	3.154
Soil B	0.328	0.179	0.645	0.129	0.105	0.355	8.197	0.891	3.217
Soil C	0.597	0.227	0.730	0.127	0.116	0.270	12.074	0.876	3.842
Soil D	0.312	0.153	0.651	0.133	0.117	0.349	9.157	0.884	3.197
Triton X-100 1%									
	*K*_sor, i_ (L/mg. min)	*K*_des, j_ (1/min)	*ϵ*_i_ (-)	*K*_sor, j_ (L/mg. min)	*K*_des, j_ (1/min)	*ϵ*_j_ (-)	*S*_max_ (mg/g_soil_)	R^2^ (-)	RMSE (mg/L)
Soil A	0.698	0.273	0.752	0.138	0.117	0.248	17.198	0.983	3.197
Soil B	0.258	0.186	0.639	0.128	0.104	0.361	7.387	0.979	3.847
Soil C	0.563	0.246	0.718	0.146	0.115	0.282	9.987	0.987	2.547
Soil D	0.298	0.169	0.624	0.129	0.106	0.376	8.215	0.991	3.363

Under competitive conditions, the results indicate minimal effects on Triton X-100 sorption–desorption, consistent with observations in the equilibrium framework. MLM results confirm that sorption and desorption coefficients ratio, nonlinearity, and maximum sorption capacities for Triton X-100 across different soils are nearly identical to those from single kinetic analyses. In contrast, under competitive conditions, glyphosate exhibits markedly different behavior compared to single-sorption–desorption scenarios, showing a significant reduction in sorption capacity, nonlinearity, and the sorption-to-desorption ratio. In the single scenario, glyphosate sorption in agricultural soils reaches 80% of the initial concentration for Soil A (exhibiting the highest sorption) and 50% for Soil B (exhibiting the lowest sorption). However, in the competitive scenario with 0.5% Triton X-100, glyphosate sorption decreases to a range of 40–65% of the initial concentration. When the Triton X-100 concentration is increased to 1%, glyphosate sorption further diminishes to 35–60%, demonstrating a consistent decreasing trend across all soil types. Based on MLM, the competitive kinetic Langmuir model estimates the total sorption capacity in agricultural soils. The results indicate that the maximum sorption capacity is even lower than that of glyphosate alone under single kinetic conditions. This finding suggests that the presence of Triton X-100 not only reduces the soil’s sorption capacity for glyphosate but also diminishes the overall sorption capacity. Furthermore, a comparison of the kinetic sorption–desorption results between single and competitive conditions reveals that in competitive scenarios, sorption occurs more slowly, with the majority of sorption not happening at a high rate similar to single sorption. This observation can be further analyzed through the sorption–desorption ratios determined by the MLM. The estimated sorption and desorption coefficients indicate that, within competitive conditions, the sorption–desorption ratio is smaller, suggesting that sorption happens at a slower rate. Triton X-100, as a surfactant, alters the physicochemical properties of the soil surface, reducing the number of available binding sites for glyphosate. This competition delays the sorption process, as glyphosate has to compete for these sites over a longer period, resulting in a slower sorption rate compared to the single-component scenario.

In competitive kinetic experiments, glyphosate sorption was not only reduced in magnitude but also slowed in rate when Triton X-100 was present. This confirms that competition delays site occupation and reduces sorption efficiency, consistent with the hypothesized mechanism of site interference (Jiang et al. 2020). Notably, Triton X-100 showed slightly enhanced kinetic uptake in the presence of glyphosate, suggesting that glyphosate may alter surface properties in a way that facilitates surfactant association. Together, these results highlight the dynamic nature of competitive sorption processes and their importance for predicting contaminant mobility.

**Fig. 8:**
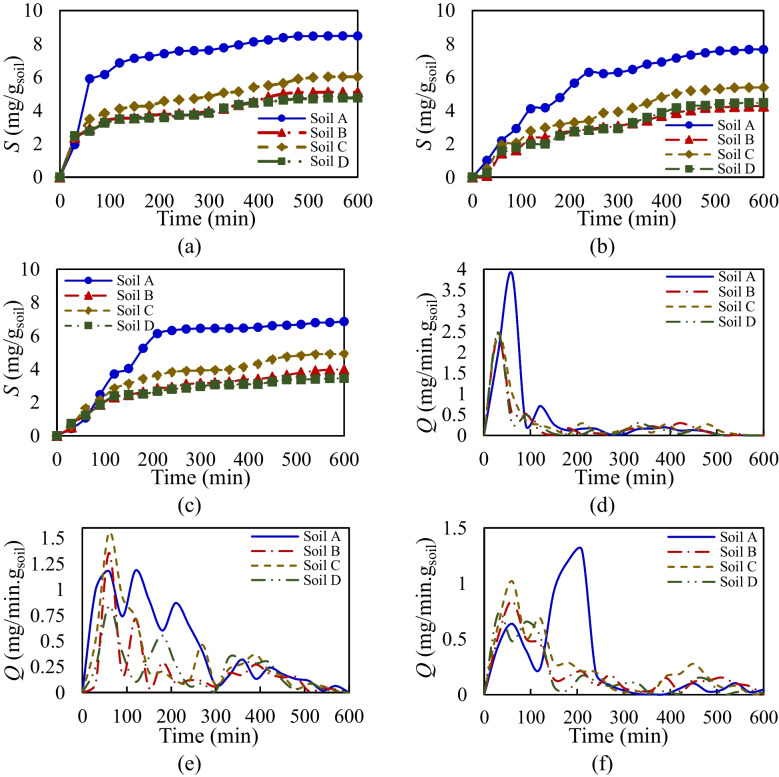
Comparing kinetic sorption values (mg/g_soil_) of glyphosate for agricultural soils in one gram of sorbent. **a** Single sorption; **b** competitive sorption 0.5%; **c** competitive sorption 1%. Comparison of sorption–desorption rates *Q* (mg/min.g_soil_) of glyphosate. **d** Single sorption; **e** competitive sorption 0.5%; **f** competitive sorption 1%

Kinetic framework enables the analysis of temporal variations in sorption–desorption mechanisms over time. Here, to clearly illustrate sorption–desorption rate variations over time, the sorption values of glyphosate in 1 g of agricultural soils under both single and competitive conditions with different concentration ratios (0.5% and 1% Triton X-100) in kinetic studies are presented in Fig. 8a–c. The results show that the overall behavior of glyphosate sorption–desorption in both single and competitive conditions can be divided into two stages: a rapid sorption phase (with low desorption rate) and a slow sorption phase (where sorption and desorption rates approach equilibrium). During the first 200 min, a large quantity of glyphosate is attached to the sorbent, indicating a high rate of sorption (rapid sorption phase). In all cases, more than 70% of pesticide sorption occurs in this rapid phase, as glyphosate molecules have ample opportunity to bind to available sorption sites on the sorbent minerals. After this phase, sorption slows, as most sorption sites are occupied, and intense competition arises between glyphosate and Triton X-100 for the remaining free sites. This competition reduces the sorption rate for both compounds. Comparing the single and competitive cases at two different concentrations reveals that the presence of Triton X-100 extends the rapid sorption phase, resulting in a slower sorption rate and a delayed equilibrium. Eventually, the system reaches its equilibrium phase, around 500 min, when sorption site saturation occurs and the difference between sorption and desorption rates becomes negligible.

Figure 8 d–f presents the variation in the sorption–desorption rate of glyphosate (*Q*, mg/min g_soil_) for both single and competitive cases (0.5% and 1% Triton X-100) across agricultural soils. Based on the sorption–desorption rate variations over time for the single scenario (Fig. 8d), the maximum sorption–desorption rate for all agricultural soils occurs during the rapid sorption phase within the first 100 min of the kinetic experiment. Soil A, exhibiting the highest sorption capacity, demonstrates the highest sorption–desorption rate, attributed to its mineral-rich composition, compared to the other agricultural soils. Comparing the sorption–desorption rate variations for the single scenario with competitive cases (0.5% and 1% Triton X-100) reveals significant differences in glyphosate’s kinetic behavior in agricultural soils. In competitive scenarios, glyphosate sorption occurs at a slower rate, indicating that Triton X-100 may enhance glyphosate’s mobility. As shown in Fig. 8e, the elevated sorption–desorption rate observed in the single scenario within the first 100 min extends across 300 min in the presence of 0.5% Triton X-100. Figure 8 f further demonstrates that increasing Triton X-100 concentration to 1% shifts and delays the high sorption rate phase by over 100 min, with a larger portion of total sorption occurring during the slower sorption phase. Moreover, in competitive cases, multiple relative peaks in sorption–desorption rates are observed during both the rapid and slow phases across all agricultural soils. This indicates that the kinetic sorption rate in competitive scenarios is slower and more prolonged, extending over a longer duration. The delayed sorption behavior is attributed to Triton X-100 modifying Glyphosate’s affinity, reducing its sorption capacity and rate in agricultural soils. These findings align with the surfactant’s intended purpose of decreasing sorption and enhancing pesticide mobility to effectively reach the root zone.

The statistical parameters and error margins presented in the “Results and discussions” section demonstrate the high accuracy and repeatability of the batch experimental data, with concentration measurements falling within a 0.95% accuracy range. Furthermore, the statistical evaluation of the estimated parameters for both single-solute and competitive sorption, under equilibrium and kinetic conditions, confirms the strong performance of the models, as indicated by low errors across repeated experimental runs.

The sorption–desorption behavior observed in the batch experiments shows trends that are broadly consistent with previous studies on glyphosate and Triton X-100 sorption in agricultural soils. Although the literature does not specifically report the competitive sorption–desorption results or kinetic stages presented in this study, for single-solute systems, the sorption coefficients and behavior obtained for glyphosate are substantially stronger than those reported for compounds such as heavy metals (Padilla et al. 2023) yet remain within the same order of magnitude as previously reported values for glyphosate (Gairhe et al. 2021). For Triton X-100, which has been less extensively studied than glyphosate, the low sorption affinity observed in our experiments aligns with findings from earlier research (Wiśniewska et al. 2021). Importantly, the present study extends these observations by providing detailed analysis under both equilibrium and kinetic conditions, revealing that competitive effects are more pronounced during the early stages of sorption, consistent with kinetic limitations reported by (Zhao et al. 2024). Furthermore, the application of the inverse modelling framework enabled quantification of parameter uncertainties, providing a level of detail rarely addressed in previous batch studies (Janetti et al. 2012).

The findings of this study have important environmental and societal implications. By investigating the competitive sorption–desorption behavior of glyphosate and Triton X-100 in agricultural soils, the study provides a better understanding of how these widely used agrochemicals interact and move through the soil system. This knowledge is critical for predicting the fate and transport of agrochemicals in the environment, particularly their potential to contaminate groundwater. The results can inform soil and water management practices, guiding the development of strategies to reduce agrochemical leaching and mitigate risks to ecosystems and human health. Ultimately, this study supports efforts toward sustainable agriculture and the protection of water resources.

## Conclusion

Based on single-competitive sorption–desorption analysis of glyphosate and Triton X-100 in equilibrium and kinetic frameworks in four agricultural soils, the following conclusions were derived:

- Findings show identical sorption capacity, nonlinearity, and sorption–desorption parameters for Triton X-100 in both single and competitive scenarios with maximum sorption capacity up to 0.2 mg/g_soil_. In contrast, the presence of Triton X-100 in competitive conditions reduces glyphosate sorption by up to 15%, while also decreasing its nonlinearity term up to 20%. Additionally, under competitive kinetic conditions, sorption and desorption rates become more closely aligned, delaying reaching equilibrium.
- Regardless of soil composition and the pesticide/surfactant concentration ratio, Triton X-100 demonstrates minimal sorption capacity (up to 0.2 mg/g_soil_) and low nonlinearity, with sorption and desorption rates remaining closely aligned under kinetic conditions. In contrast, glyphosate exhibits a strong sorption affinity (up to 27 mg/g_soil_), significantly influenced by minerals such as albite, muscovite, kaolinite, and illite, which can enhance sorption capacity by up to 45%. Furthermore, increasing the concentration of Triton X-100 from 0.5 to 2% results in an additional 10% reduction in glyphosate sorption capacity and further delays the attainment of equilibrium within the kinetic framework.
- In both equilibrium and kinetic frameworks, the nonlinear Freundlich and Langmuir isotherms more accurately represent the sorption–desorption behavior compared to the linear model, which fails to capture the system’s dynamic nature. Among these models, the kinetic Langmuir model exhibits the best performance, effectively estimating the maximum sorption capacity while accounting for temporal variations in concentration and sorption–desorption processes, as confirmed by the MLM analysis.
- Kinetic analysis indicates a two-stage sorption–desorption process for glyphosate under both single and competitive conditions: a rapid phase (~ 70% of total sorption with minimal desorption) followed by a slower phase as equilibrium is approached. The presence of Triton X-100, especially at higher concentrations, is associated with a longer rapid phase and delayed equilibrium.
- Peak sorption–desorption rates (*Q*) for glyphosate occur within the first 100 min under single-sorption conditions. In competitive scenarios, Triton X-100 shifts this peak to around 300 min and increases sorption during the slower phase, with multiple relative peaks observed across soils.

Future studies can leverage the MLM model and associated sorption–desorption parameters to simulate large-scale agricultural systems, capturing the competitive sorption of pesticides and surfactants. Building on the findings of this study, future research should explore the competitive sorption under more variable environmental conditions, including changes in pH, temperature, and ionic strength. Incorporating additional organic and inorganic co-contaminants could further clarify multi-sorbate interactions in complex systems. Moreover, evaluating a broader range of soil types with varying organic matter and texture can enhance the generalizability of the proposed method and support its application in risk assessment and environmental management frameworks.

## Figures and Tables

**Fig. 9:**
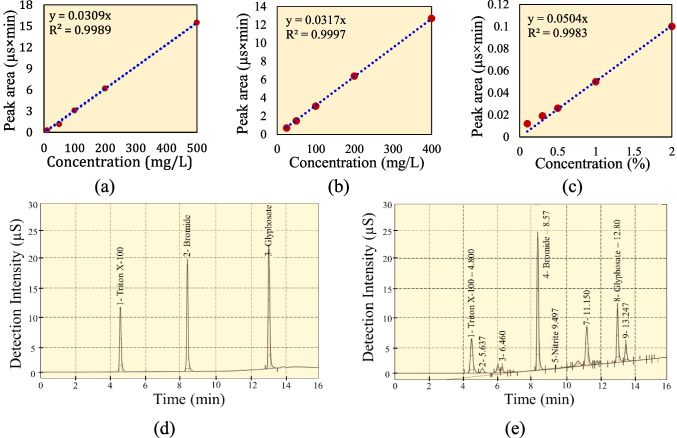
ICMS Calibration curves for **a** bromide, **b** glyphosate, and **d** Triton X-100. Detection results for **d** prepared solution and **e** final samples after experiment

**Fig. 10:**
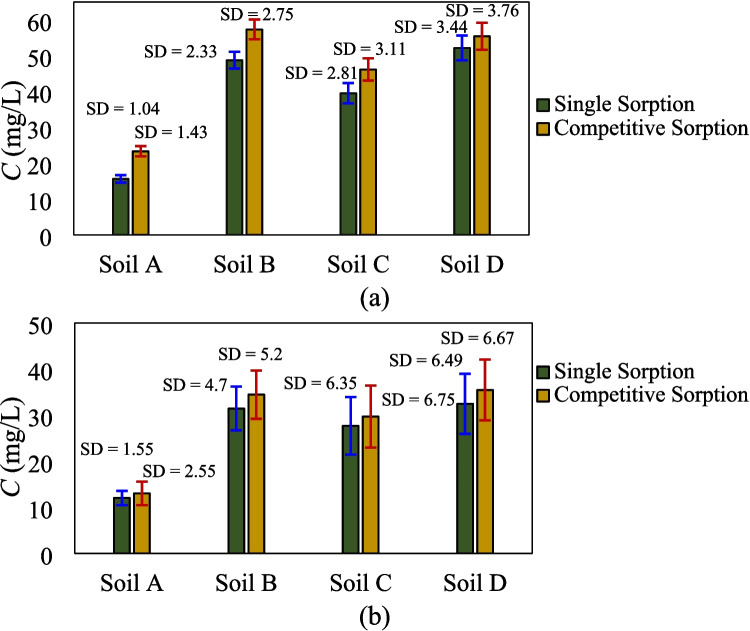
Descriptive error bars for experimental concentration measurements for glyphosate in agricultural soils. **a** Equilibrium; **b** kinetic batch data. The columns denote the concentration mean value, and the bars on the columns represent the SD

## Data Availability

The experimental data and numerical findings are fully presented in the paper.

## Appendix 1

Concentration measurement of glyphosate because of high polarity, low volatility, and lack of chromophore is challenging. Some studies proposed using high-performance liquid chromatography (HPLC) approach (Valle et al. 2019). However, according to glyphosate’s chemical structure, this method needs derivatization, which is complex and time-consuming. IC-MS is an efficient for glyphosate detection, according to chemical ionic metabolites. The IC-MS system has been calibrated for the studied chemicals using standard solution samples in various concentrations. As presented in Fig. 9a–c, the calibration accuracy achieved for bromide, glyphosate, and Triton X-100 is exceptionally high, with values of 0.9994, 0.9999, and 0.9983, respectively for the coefficient of determination. Each calibration sample was analyzed in three replicates by the IC-MS. The limit of quantification of the developed method is concentrations lower than 1 µg/L. Figure 9d–e show the detected bromide, glyphosate, and Triton X-100 in prepared samples and final samples including compositions from soil samples. According to the developed method IC-MS, Triton X-100 is the first detected compound at 5.026 min. Then, bromide and glyphosate are detected at 8.57 and 12.80 min.

## Appendix 2

Measurement errors are evaluated to analyze the reliability of the experimental data generated for single-competitive sorption. Each experiment for batch equilibrium-kinetic sorption–desorption in single and competitive systems is repeated three times to evaluate the measurement errors. In Fig. 10, descriptive error bars for experimental concentration measurements for glyphosate in four agricultural soil samples are presented. Results depict good reliability on data according to the negligible standard deviation (SD).

## Appendix 3

**Table 8:** Estimated parameters for single and competitive sorption models evaluated by the developed MLM and the reference study (Janetti et al. 2012)

Single	Bet Dagan Soil									Yatir Soil								
Isotherm	Cu				Zn					Cu				Zn				
Freundlich	(Janetti et al. 2012).		Present study		(Janetti et al. 2012)			Present study		(Janetti et al. 2012)		Present study		(Janetti et al. 2012)			Present study	
K_F_ (L^m^/mg^1-m^. g_soil_)	0.990		0.983		0.580			0.578		2.220		2.380		2.280			2.259	
*m *(-)	0.260		0.262		0.390			0.379		0. 180		0.201		0.290			0.310	
Langmuir																		
K_L_(L/mg)	0.280		0.240		0.070			0.072		0.400		0.385		0.080			0.076	
S_max_(mg/g_soil_)	2.860		2.780		3.680			3.670		11.140		10.94		11.690			11.85	
Binary	Cu and Zn									Cu and Zn								
Extended Langmuir	(Janetti et al. 2012)						Present study			(Janetti et al. 2012)						Present study		
K_Li_(L/mg)	0.05						0.06			0.03						0.04		
K_Lj_(L/mg)	0.02						0.03			0.02						0.03		
S_max_(mg/g_soil_)	5.36						5.41			12.45						12.67		
